# Rational discovery of antimetastatic agents targeting the intrinsically disordered region of MBD2

**DOI:** 10.1126/sciadv.aav9810

**Published:** 2019-11-20

**Authors:** Min Young Kim, Insung Na, Ji Sook Kim, Seung Han Son, Sungwoo Choi, Seol Eui Lee, Ji-Hun Kim, Kiseok Jang, Gil Alterovitz, Yu Chen, Arjan van der Vaart, Hyung-Sik Won, Vladimir N. Uversky, Chul Geun Kim

**Affiliations:** 1 Department of Life Science and Research Institute for Natural Sciences, Hanyang University, Seoul 04763, Korea.; 2 Department of Molecular Medicine, Morsani College of Medicine, University of South Florida, Tampa, FL 33612, USA.; 3 Department of Pathology, Hanyang University College of Medicine, Seoul 04763, Korea.; 4 College of Pharmacy, Chungbuk National University, Cheongju, Chungbuk 28160, Korea.; 5 Boston Children's Hospital/Harvard Medical School, Boston, MA 02115, USA.; 6 Department of Chemistry, University of South Florida, Tampa, FL 33620, USA.; 7 Department of Biotechnology, Konkuk University, Chungju, Chungbuk 27478, Korea.; 8 Institute for Biological Instrumentation of the Russian Academy of Sciences, Pushchino, Moscow Region 142290, Russia.

## Abstract

Although intrinsically disordered protein regions (IDPRs) are commonly engaged in promiscuous protein-protein interactions (PPIs), using them as drug targets is challenging due to their extreme structural flexibility. We report a rational discovery of inhibitors targeting an IDPR of MBD2 that undergoes disorder-to-order transition upon PPI and is critical for the regulation of the Mi-2/NuRD chromatin remodeling complex (CRC). Computational biology was essential for identifying target site, searching for promising leads, and assessing their binding feasibility and off-target probability. Molecular action of selected leads inhibiting the targeted PPI of MBD2 was validated in vitro and in cell, followed by confirming their inhibitory effects on the epithelial-mesenchymal transition of various cancer cells. Identified lead compounds appeared to potently inhibit cancer metastasis in a murine xenograft tumor model. These results constitute a pioneering example of rationally discovered IDPR-targeting agents and suggest Mi-2/NuRD CRC and/or MBD2 as a promising target for treating cancer metastasis.

## Teaser

The intrinsically disordered region of MBD2 is a promising target for treating cancer metastasis.

## INTRODUCTION

Although at least 650,000 protein-protein interactions (PPIs) might occur in humans, only one PPI inhibitor has been approved for clinical use to treat cancers (*1*), suggesting that the field of PPI inhibitors remains largely unexplored. A variety of proteins and their PPIs have emerged as prospective drug targets to treat tumors because of the extreme heterogeneity and plasticity of cancer (*2*, *3*). Ligands with the potential of binding to a specific site of a target protein with known structure can be efficiently identified by virtual screening. However, the structural plasticity of target proteins usually works against yielding an effective drug candidate. For example, selected compound treatment of cells/organisms often fails to elicit the anticipated effects due to in vivo structural alterations of the target protein caused by various posttranslational modifications (PTMs) and/or unanticipated interactions of the compound and/or its target protein with other molecules (*4*, *5*). Furthermore, many critical proteins regulating various biological processes do not have unique structures as a whole or in some functionally important regions (*6*, *7*). Structures of these intrinsically disordered proteins (IDPs) or IDP regions (IDPRs) are extremely dynamic, depending on the environment, and might change during function (*4*, *8*). Many signaling IDPs/IDPRs undergo characteristic disorder-to-order transitions (DOTs) upon interactions with specific binding partners and/or through PTMs (*9*, *10*). Targeting the IDPs/IDPRs capable of DOT is generally considered an attractive but challenging task for developing anti-PPI inhibitors. In this regard, a recently identified small-molecule compound, 10058-F4, serves as a pioneering success of anti-PPI inhibitor that binds to an IDPR of c-Myc undergoing a DOT upon binding to its partner Max (*11*, *12*). 10058-F4 was discovered by a random screening using a yeast two-hybrid system (*11*), followed by experimental identification of its specific binding site (residues 402 to 412 of c-Myc) as an IDPR. Drug leads like 10058-F4 targeting IDPs/IDPRs cannot be found by conventional computational methods that rely on fixed conformations, such as crystallographic structures of target proteins. No computer-aided drug discovery platform is currently available for the systematic exploration of IDPRs as potential drug-target sites (*3*).

**Fig. 1:**
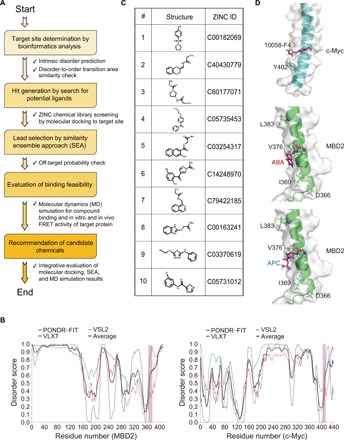
In silico discovery of the MBD2 IDPR-targeting ligands. (**A**) Flow chart describing the computational process of ligand discovery. (**B**) Evaluation of the intrinsic disorder propensity of MBD2 (left) and c-Myc (right); disorder scores 1 and 0 mean fully disordered and fully ordered residues, respectively. Pink bars show positions of the determined DOT sites embedded in residues 360 to 393 for MBD2 and 395 to 430 for c-Myc. (**C**) Chemical structures of the top 10 compounds showing the most favorable binding to the MBD2 target site in the molecular docking screening of ZINC chemical library. (**D**) Representative structures of protein-ligand complexes obtained from the molecular docking results (original data file 1 for PDB coordinates): 10058-F4:c-Myc_402_ (top; control experiment), ABA:MBD2_369_ (middle), and APC:MBD2_369_ (bottom).

To fill this gap, we developed a novel platform for the discovery of drug leads based on molecular docking and molecular dynamics (MD) simulations of the DOT-associated IDPRs of target proteins. Figure 1A describes this protocol. First, intrinsic disorder predispositions of drug-target proteins are analyzed, and potential disorder-based binding regions that can undergo DOTs are evaluated. A search of the protein structure database [Protein Data Bank (PDB)] is also performed to identify known PPIs and DOTs. Once the potential drug-target sites (DOT-based PPI regions) are determined, the corresponding structures retrieved from the PDB are used for molecular docking with druggable compounds from the ZINC compound library (*13*). Together with the docking scores, off-target probabilities assessed by the similarity ensemble approach (SEA) (*14*–*16*) analysis are also considered for selection of lead compounds from the molecular-docked hit compounds. Last, prospected candidate compounds are suggested via MD simulations that evaluate the mode and efficiency of the compound binding.

The feasibility of the proposed approach was validated in this study by targeting an IDPR of MBD2 that undergoes a DOT upon association with its binding partner p66α for the integration of the Mi-2/NuRD chromatin remodeling complex (CRC). The integrated Mi-2/NuRD CRC includes one CHD (either CHD3 or CHD4), one HDAC (HDAC1 or HDAC2), two DOC1, three MTA (MTA1, MTA2, and MTA3), six RbAp46/48, two p66 (p66α or p66β), and one MBD (MBD2 or MBD3) molecules (*17*, *18*), where the molecular interaction of MBD2 with p66α critically mediates the proper assembly of CRC (*17*, *19*). This CRC performs an important epigenetic function in normal development and differentiation by suppressing gene expression by binding directly to the DNA methylation sites and to the DNA methyltransferases (*20*, *21*).

CRC also contributes to the development of human diseases, including cancer (*22*, *23*); for example, the epigenetic regulation by Mi-2/NuRD CRC includes multiple tumor suppressor genes (*23*, *24*), and several CRC components, including MBD2, were also observed to be oncogenic and/or closely correlated with the aggressiveness of several cancers (*23*, *25*, *26*). In particular, the function of Mi-2/NuRD CRC is known to be associated with the cellular process of epithelial-mesenchymal transition (EMT; the conversion of adhesive epithelial cells into migratory, invasive mesenchymal cells) that drives wound healing and cell migration and invasion (*27*, *28*). In cancer, EMT necessarily mediates the metastasis of cancers and thus also enables carcinoma cells to acquire cancer stem cell (CSC) properties, malignancy-associated traits, and drug resistance (*29*–*31*). Given that the metastasis is responsible for more than 90% of contemporary cancer deaths and yet no marketed antimetastatic drug is currently available (*32*), developing these drugs to target the cancer spreading is an essential and urgent task for oncological therapy. In this context, functional inhibition of CRC or modulation of its individual components might serve as a novel strategy for effective anticancer therapy to prevent the progression of cancer to metastatic stage. In particular, it has been observed that down-regulation of MBD2 and/or p66α, which triggered derepression of epithelial regulators via epigenetic reprogramming of the Mi-2/NuRD CRC into the MBD2-free or disentangled CRC, resulted in promoted epithelial differentiation and loss of tumor-initiating ability. Therefore, targeting MBD2 specifically at its IDPR would be a promising approach to the development of antimetastatic agents by inhibiting its DOT-based PPI with p66α that is essential for the integration of CRC and thus for its critical function in EMT. In addition, no noticeable adverse effects displayed by MBD2 inhibitors can be expected from the fact that down-regulation of *MBD2* expression is essential for normal cell differentiation (*33*), and yet, *MBD2* knockout (*MBD2^−/−^*) mice exhibit normal survival and reproduction (*34*).

Hence, in this study, the MBD2 IDPR and its DOT-based interaction with p66α for the CRC integration were selected as a highly promising target system to evaluate the efficiency of our platform for rational drug discovery. Using this novel approach, we identified two small-molecule compounds capable of inhibiting the PPI of MBD2 and thereby efficiently suppressing the cancer metastatic potentials. In vivo efficacy of both leads in inhibiting cancer metastasis was also evident in a murine xenograft tumor model. Therefore, our novel method renders IDPRs available for rational discovery of anticancer drugs targeting DOT-based PPIs. In particular, the identified compounds provide a basis for the development of previously unidentified inhibitors capable of controlling metastasis of various carcinomas.

## RESULTS

### In silico analysis for determination of target site and search for potential ligands

As our study was inspired by the discovery of 10058-F4, which binds to the c-Myc IDPR to inhibit its DOT for interaction with Max (*11*, *12*), we compared the PPI site of MBD2 with that of c-Myc using our computational platform. Sequence analysis (see fig. S1 for sequence and structure information) revealed that disorder profiles of the PPI site of MBD2 (residues 360 to 393 for p66α interaction) (*17*, *35*) closely resembled that of c-Myc (residues 400 to 434 for Max interaction) (*36*, *37*) (Fig. 1B), characterized by a positive slope in its disorder profile. As both MBD2 and c-Myc are folded in complexes with their cognate partners (p66α and Max, respectively) (*17*, *35*, *37*), this analysis suggests that the PPI sites of MBD2 and c-Myc could undergo a similar type of DOT upon complex formation.

Subsequently, a nuclear magnetic resonance (NMR) ensemble structure of MBD2_360–393_ in its complex with p66α_138–178_ (PDB ID: 2L2L) was retrieved, and the lowest-energy conformation of the ensemble was extracted for molecular docking analysis using the four residues (D_366_, I_369_, V_376_, and L_383_) of MBD2_360–393_ engaged in the coiled-coil interaction, with p66α (*35*) as the initial target site in the molecular docking. From the molecular docking–based virtual screening of 2 × 10^6^ chemical compounds in the ZINC library, 10 promising compounds (compounds #1 to #10 in Fig. 1C) capable of interaction with MBD2 at the designated target site were selected. As a control, the Y_402_-targeted molecular docking of 10058-F4 to c-Myc_395–430_ (Fig. 1D; note that the key residue for the c-Myc interaction with 10058-F4 is Y_402_) (*35*) was compared with the MBD2_360–393_ docking of the 10 selected hit compounds (table S1). The MBD2_369_-targeted docking of two compounds {compounds #2 and #3 in Fig. 1D named herein ABA [2-amino-*N*-(2,3-dihydro-benzo[1,4]dioxin-2-ylmethyl)-acetamide] and APC [3-(2-amino-acetylamino)-pyrrolidine-1-carboxylic acid tert-butyl ester], respectively} was found as the most favorable. In ABA:MBD2_369_ and APC:MBD2_369_ dockings, these compounds formed three intermolecular hydrogen bonds and had relatively low DOCK scores (−35.2 and −33.3 kcal mol^−1^, respectively) of the DOCK binding. These binding features could be compared favorably with those of the 10058-F4:c-Myc_402_ docking, which showed the DOCK score of −6.77 kcal mol^−1^ and just one intermolecular hydrogen bond (table S1).

### Selection of lead compounds by in silico assessment of off-target probability and activity test in cells

**Fig. 2:**
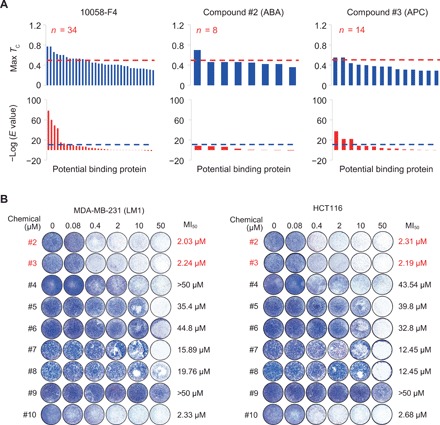
Lead selection from hit compounds. (**A**) Computational analysis for off-target probabilities of the 10058-F4 (control experiment) and two selected lead compounds (ABA and APC). Max *T*_c_ and *E* value of the predicted binding are plotted for the *n* (number of potential targets predicted) off-target candidates yielded from SEA using 2060 human proteins in the database. See fig. S2 for the other hit compounds. (**B**) Cell migration inhibition by the hit compounds. The LM1 and HCT116 cancer cells were fixed and stained after 48 hours of Transwell migration in the presence of indicated concentrations of individual compounds, followed by counting the number of migrated cells (*n* = 2) to yield MI_50_ value.

Concerning the potential side effects of the selected hit compounds, their off-target probabilities were assessed by the SEA analysis (*14*, *16*), which has served as an eminent bioinformatics resource aiding in target identification for drug development by profiling multiple protein targets of chemical compounds as probes (*15*). For this analysis, the c-Myc inhibitor 10058-F4 and two anticancer drugs, imatinib (Gleevec) and sorafenib (Nexavar), were also compared as controls, and 2060 human proteins in the database were searched as potential targets. Given that a significant binding is feasible when both the Max *T*_c_ value more than 0.5 and *E* value lower than 10^−10^ are relevant, no suggestible off-target was predicted for 7 of the 10 hit compounds including both ABA and APC, whereas four proteins were found as the probable 10058-F4 targets (Fig. 2A and table S2). Two of the other compounds also showed a small number of putative off-target proteins (six and two proteins for compounds #4 and #10, respectively), whereas 35 and 26 targets were suggested for imatinib and sorafenib, respectively (fig. S2A and table S2). Therefore, we screened nine compounds with low off-target probability for cellular activity dysregulating MBD2. In particular, the cell migration assay was used for this preliminary test of the compounds on the basis of the previous observation that knockdown of MBD2 in cancer cell lines resulted in decreased migration of the cells. The result implicated most of the hit compounds in actual suppression of the migration of breast adenocarcinoma MDA-MB-231 (LM1) and colorectal carcinoma HCT116 cells (Fig. 2B and fig. S2B). In particular, ABA (compound #2) and APC (compound #3), which accomplished the most favorable target binding in the aforementioned molecular docking experiments, also showed the least MI_50_ (concentration for half-inhibition of cell migration) values. Therefore, these two molecules were selected as lead compounds for subsequent evaluation in detail.

### In silico analysis of target binding for selected lead compounds

**Fig. 3:**
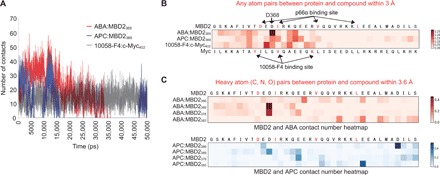
In silico analysis of the lead compound binding to target site. (**A**) Time-course alterations of the number of intermolecular contacts within 3 Å cutoff in MD simulations. (**B**) Heatmap describing the number of simulated compound-protein contacts during 50-ns trajectory for individual residues. Each value of a number of contacts was normalized by dividing it by the total number of contacts in each simulation. The already-known critical residues for PPI are shown in darker red. (**C**) Heatmap of the intermolecular heavy atom contacts between the lead compounds and target proteins during 50-ns trajectory. Number of contacts was normalized by the total number of contacts in each simulation. MBD2 N-terminal two residues, G and S, were from the NMR structure (PDB ID: 2L2L). MBD2 sequence starts from K360, after G, and S.

To assess target-binding feasibility and mode of binding of the two selected leads, we conducted MD simulation using the structures resulting from the ABA:MBD2_369_, APC:MBD2_369_, and 10058-F4:c-Myc_402_ docking (Fig. 1D) as starting points. In 50-ns MD trajectories, the number of the compound-protein contacts (Fig. 3A) and the compound-protein interaction energies (fig. S3A) over time were steady for 10058-F4:c-Myc_402_ but showed noticeable fluctuations for ABA:MBD2_369_ and APC:MBD2_369_, particularly during the first half of the simulation period, suggesting that the binding of ABA or APC to MBD2_360–393_ might be less persistent than the 10058-F4–c-Myc_395–430_ interaction. However, heatmaps representing intermolecular contacts for individual residues (Fig. 3B) indicated frequent contacts of the ABA/APC–MBD2_360–393_ interaction comparable to that of the 10058-F4–c-Myc_395–430_ interaction. In particular, the highest contact density value at the most contacted residue (D368 contact) in the ABA:MBD2_369_ trajectory was higher than that (L404 contact) in the 10058-F4:c-Myc_402_ trajectory, suggesting stronger binding. Next, MD simulations for the ligand:MBD2_360–393_ complex were extended to include D_366_-, V_376_-, and L_383_-targeted docking (Fig. 3C). Consistent with the ABA:MBD2_369_ trajectory, D_368_ was the most contacted residue in the heatmaps for heavy atom contacts of the ABA:MBD2_376_ trajectory, although no preferential contact was found in the other ABA:MBD2_360–393_ trajectories and in the APC:MBD2_360–393_ MD simulation sets. Collectively, the MD simulation indicated that the actual binding of ABA and APC to MBD2_360–393_ would be as promising as the 10058-F4 binding to c-Myc_395–430_, although detailed interaction modes can be different between the two compounds. Therefore, it was subsequently examined whether the targeted binding of the compounds to MBD2 would influence specific PPI of the protein.

### Inhibition of the DOT-mediated PPI of MBD2 by the lead compounds

It has been suggested that 10058-F4 evokes a local conformational change (*36*) or conformational equilibrium shift (*38*, *39*) of the c-Myc IDPR at its binding sites, and this small but significant alteration is critically involved in the functional inhibition of the DOT-mediated PPI of c-Myc with Max. Detailed inspection of the MD simulation results suggested that the MBD2 IDPR could also undergo a local conformational perturbation upon the binding of ABA and APC. For instance, in the ABA:MBD2_369_ and APC:MBD2_369_ trajectories, both Φ and Ψ backbone torsion angles of the most contacted residue (D_368_) in the compound-contacting states were significantly (*t* test, *P* < 0.05) different from those in the noncontacting states (fig. S3B). The compound-bound conformation also appeared to be different between ABA and APC, as the D_368_ Ψ angles in the compound-contacting states were significantly different in between ABA:MBD2_369_ and APC:MBD2_369_ trajectories, although Φ angle differences were not significant (*t* test, *P* = 0.574). Therefore, to further analyze the possible conformational perturbation, we compared the compound-bound ABA:MBD2_369_ and APC:MBD2_369_ trajectories with the apo-MBD2 and p66α-MBD2 trajectories (fig. S3C). The backbone root mean square fluctuation values of individual residues (fig. S3D) showed that apo-MBD2 underwent stronger backbone fluctuations than compound- or p66α_138–178_-bound MBD2_360–393_. This reflects the structural instability of MBD2_360–393_ in the absence of bound molecules (or, conversely, DOT upon complex formation). Notably, the backbone fluctuation was also different between compound- and p66α_138–178_-bound MBD2_360–393_, especially at the p66α_138–178_-contacting D_366_ and I_369_ residues, reflecting the compound-specific local conformational perturbation in MBD2_360–393_. The presence of this compound-specific perturbation was also obvious from torsion angle distributions of the p66α_138–178_-interacting D_366_, I_369_, V_376_, and L_383_ residues (fig. S3E), as the backbone Φ/Ψ torsion angles in both ABA:MBD2_369_ and APC:MBD2_369_ trajectories were different from those in apo-MBD2 and MBD2-p66α (tables S3 and S4). In addition, comparison between ABA:MBD2_369_ and APC:MBD2_369_ MD trajectories revealed that the two compounds likely evoked different local conformational changes at the p66α_138–178_-interacting residues of MBD2. In particular, significant difference in Ψ of I_369_ and Φ/Ψ of V_376_ and L_383_ (table S4), which is distinguished from the similarity in Φ/Ψ of D_366_ and Φ of I_369_, suggested that I_369_ served as a turning point for the observed torsion angle differences more evident in its C-terminal region from I_369_. Collectively, comparative MD simulations of MBD2_360–393_ in different states (apo-, compound-, and p66α_138–178_-bound) suggested the compound-specific induction of local conformational perturbation of MBD2, especially at its p66α-interacting site, which would most likely interfere with the MBD2-p66α interaction. Therefore, we next examined whether these leads can actually inhibit the PPI of MBD2, with p66α both in vitro and in cell, by fluorescence resonance energy transfer (FRET) and co-immunoprecipitation (co-IP) assay.

**Fig. 4:**
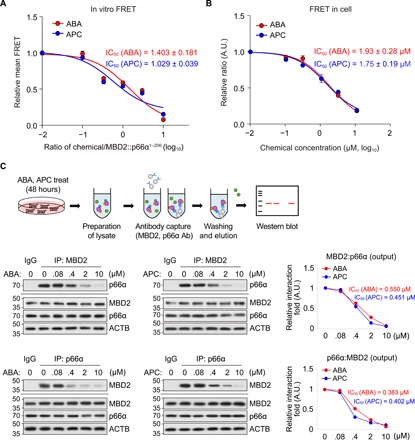
Two lead compounds efficiently disrupt MBD2 interaction with p66α in vitro and in cell. (**A**) Inhibition of in vitro FRET dynamics of MBD2 interaction with p66α by ABA and APC. Relative mean FRET values for the corresponding ratios of chemical concentration over MBD2::p66α^1–206^ were plotted. See fig. S4A for the original data. *n* = 3. (**B**) Inhibition of FRET dynamics of MBD2 interaction with p66α by ABA and APC in cells. Quantified FRET activities of mock- and compound-treated samples were obtained, and the relative FRET ratios for compounds were calculated by FRET_comp_/FRET_mock_ (see Materials and Methods). See also fig. S4B for representative immunofluorescence microscopic photos of cells. *n* = 2. (**C**) Dose-dependent suppression of the endogenous MBD2-p66α association by the ABA and APC compounds revealed by in vivo co-IP. Relative fold changes of MBD2 interaction with p66α (right) were obtained by the quantification of immunoblots (left). Data (means ± SD) in (A) and (B) were analyzed using Student’s *t* test. Ab, antibody; IgG, immunoglobulin G.

As the coiled-coil interaction between MBD2 and p66α occurs in an antiparallel fashion (*17*), MBD2 was fused with a FRET acceptor protein dTomato at its N terminus, whereas the FRET donor enhanced yellow fluorescent protein (eYFP) was C-terminally fused to p66α^1–206^ (*33*) for in vitro FRET. Unfortunately, the full-length p66α was not available for the in vitro FRET studies due to the inclusion body formation in the *Escherichia coli* system for the recombinant production. The in vitro FRET result evidenced that both ABA and APC efficiently interfere with the MBD2-p66α interaction by provoking significant reduction of FRET, which, at 1 to 1.5 equimolar concentrations of the compounds, reached half of the value recorded for the MBD2-p66α^1–206^ complex (Fig. 4A and fig. S4A). The FRET analysis in 293T cells by transient cotransfection of eYFP-MBD2 and mCherry-p66α expression constructs also showed the noticeable FRET reduction, which was dependent on the concentrations of the compounds used for the treatment (Fig. 4B and fig. S4B). Furthermore, the half maximal inhibitory concentration (IC_50_) values determined in this in-cell FRET experiments (1.93 and 1.75 μM for ABA and APC, respectively; see Fig. 4B) were in good agreement with the MI_50_ values determined in the migration assay (2.03 and 2.24 μM for ABA and APC, respectively; Fig. 2B). Last, the results of the co-IP assay to capture the endogenous MBD2-p66α complex corroborated the fact that ABA and APC inhibit the MBD2-p66α association with the submicromolar IC_50_ (Fig. 4C). Therefore, as the interruption of the MBD2-p66α interaction is anticipated to result in the prevention of the proper assembly of Mi-2/NuRD CRC, we subjected the compounds to an in-depth evaluation of biological activities targeting the function of Mi-2/NuRD CRC in cellular EMT and thereby in cancer metastasis.

### Specific action of the two identified leads suppressing metastatic potentials of cancer cells

**Fig. 5:**
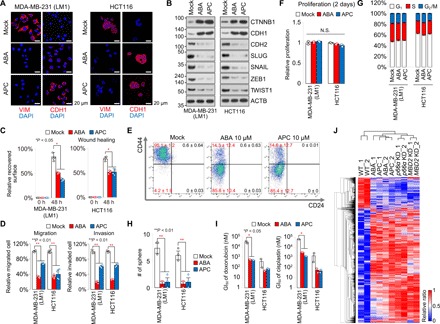
Induction of a MET on treatment of mesenchymal type of cancer cells with ABA or APC. (**A**) Representative images showing immunofluorescent signals for VIM or CDH1 (red) and 4′,6-diamidino-2-phenylindole (DAPI) (blue) in LM1 (left) and HCT116 (right) cells treated with 10 μM ABA or APC. Photo credit: S.H.S., Hanyang University. (**B**) Immunoblots showing the expression levels of EMT markers 48 hours after compound (10 μM) treatment. ACTB was used as a loading control. A.U., arbitrary units. (**C**) Effects on wound healing, estimated by the recovered surface areas of scraped cell monolayers, 48 hours after treatment with 10 μM ABA or APC. *n* = 4. (**D**) ABA and APC (10 μM) impact on cell migration (left) and invasion (right) represented by the number of migrated and Matrigel-invaded cells in Transwell plates 48 hours following compound treatments. *n* = 3. (**E**) Relative proliferation rates quantified by 3-(4,5-dimethylthiazol-2-yl)-2,5-diphenyltetrazolium bromide (MTT) assay after 2 days. Cells were treated with 10 μM ABA or APC. *n* = 2. (**F**) Cell cycle analysis by fluorescence-activated cell sorter (FACS). Cells were treated with 10 μM ABA or APC. *n* = 2. (**G**) Number of spheres counted by the naked eye after 5 days. Cells were treated with 10 μM ABA or APC. *n* = 3. (**H**) Representative cell population images for the stem-like CD44^hi^ profile of the ABA- or APC-treated LM1 cells analyzed by FACS. Data from one experiment are shown as averages of two technical replicates. (**I**) Sensitivity to doxorubicin (left) and cisplatin (right) of the 10 μM ABA- or APC-treated cells quantified by MTT assay. *n* = 2. (**J**) Heatmap of mRNA-Seq data, which demonstrates similarity in gene expression between ABA- or APC-treated cells and MBD2 or p66α knockdown LM1 cells. Data (means ± SD) in (E) to (I) were analyzed using Student’s *t* test. **P < 0.01 and *P < 0.05.

The cellular EMT process that drives cell migration and invasion is critical not only for wound healing but also for cancer metastasis, including promotion of CSC and drug-resistant properties of cancer cells (*29*–*31*). As we have previously observed that the MBD2 and/or p66α down-regulation in cancer cell lines resulted in the depressed EMT and conversely promoted epithelial differentiation, we reasoned that disrupted PPI between MBD2 and p66α by the ABA and APC compounds could result in suppression of metastatic potentials of cancer cells by regulating the Mi-2/NuRD CRC–mediated EMT. In agreement with these hypotheses, in mesenchymal type of cancer cells (triple-negative and basal-type breast cancers and aggressive colon cancers) treated with ABA or APC, the increased levels of epithelial markers (CDH1 and CTNNB1) were appreciable, whereas the mesenchymal marker (VIM, SNAIL, SLUG, and CDH2) expressions were suppressed. On the other hand, such an alteration indicative of mesenchymal-epithelial transition (MET) was not apparent in the epithelial cancer cells (luminal breast cancers and less aggressive colon cancer) (Fig. 5, A and B, and fig. S5A). Subsequent analyses confirmed that the compounds suppressed wound healing and migration/invasion abilities of the cancer cells (Fig. 5, C and D, and fig. S5B). In addition, flow cytometric measurements of the cell surface markers CD44 and CD24 indicated that the LM1 cells of the stem-like phenotype (CD44^hi^/CD24^lo^) were switched over to the nonstem phenotype (CD44^lo^/CD24^lo^) by the compound treatments (Fig. 5E), although the compounds did not induce significant alterations in the proliferation rates and cell cycle progression of the cells tested (Fig. 5, F and G, and fig. S5, C and D). Furthermore, the compound-treated cancer cells showed reduced capability of mammosphere formation (Fig. 5H and fig. S5E), thereby resulting in enhanced susceptibility of the cells to chemotherapeutic drugs including doxorubicin and cisplatin (Fig. 5I and fig. S5F). Last, mRNA sequencing (mRNA-Seq) results showed that global gene expression profiles of the ABA- or APC-treated cells were highly comparable to those of MBD2- or p66α-knockdown cells but markedly discriminated from the profiles of nontreated wild-type cells (Fig. 5J), supporting no significant off-target effects as initially predicted by SEA (Fig. 2A). Together, these observations established antimetastatic activity of the lead compounds, ABA and APC, by demonstrating that the compounds actioned so specifically on the MBD2-p66α PPI system that the EMT process was efficiently modulated to induce transition of CSC-like cells from a mesenchymal-like state to a bona fide epithelial state.

### Verification of antimetastatic efficacy of the two selected lead compounds

**Fig. 6:**
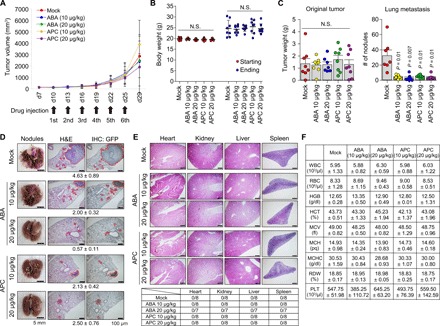
In vivo antimetastatic efficacy of ABA and APC. (**A**) Estimated volume (means ± SEM; *P* value for significance test by ANOVA) of original tumor developed during the experimental period with and without the drug administration. *n* = 8 for each group. (**B**) Body weights of mice monitored at the starting and ending point of experiment. (**C**) Effects of the compound administration on the xenograft tumor and its metastasis. Estimated tumor weights are presented for the original tumors, whereas the number of nodules developed by lung metastasis is plotted. (**D**) Representative photographs for lung nodules acquired 29 days after injection of the LM1 cells. Images of metastasized lung tissue sections illustrated by hematoxylin and eosin (H&E) staining and GFP immunohistochemistry (IHC). Yellow arrowhead represents the tumor nodule, and red dotted area indicates the tumor region. Numbers below the H&E-stained tissue sections indicate the average number of tumor nodules in all mice of the same group. Photo credit: M.Y.K. and S.C., Hanyang University. (**E**) Representative images of H&E-stained tissue sections for the major organs derived from the xenograft NOD-Prkdc^scid^ IL2rg^−/−^ (NPG) mice after completion of the metastasis inhibition tests with the ABA and APC administration (top). Histological scoring (tumor-bearing mice/total mice) for the H&E-stained major organs of the xenograft mice (bottom). Scale bars, 500 μm. Photo credit: M.Y.K. and S.C., Hanyang University. (**F**) CBC analysis of the ABA- and APC-treated xenograft mice. WBC, white blood cell count; RBC, red blood cell count; HGB, hemoglobin; HCT, hematocrit; MCV, mean corpuscular volume; MCH, mean corpuscular hemoglobin; MCHC, mean corpuscular hemoglobin concentration; RDW, red cell distribution width; PLT, platelet count; N.S., not significant. Data (means ± SD) in (B) to (D) and (G) were analyzed using Student’s *t* test.

Antimetastatic efficacy of the two selected lead compounds in vivo was analyzed using xenograft mice transplanted with the LM1 cells, which were chosen for its potent ability to readily metastasize to lung in mice (*40*). Here, ABA (10 μg kg^−1^) and APC (20 μg kg^−1^) compounds were administered by intravenous injection six times every 3 days from day 10 after the subcutaneous injection of the green fluorescent protein (GFP)–tagged LM1 cells, followed by sacrifice of the mice (after 4 days of the last administration) for subsequent analysis of tumors (Fig. 6, A and B). Notably, although growth inhibition of original tumor was not significant (Fig. 6, A, C, and D), both ABA and APC compounds exhibited a potent inhibition of the cancer metastasis to lung (represented by the number of nodules developed in lung; Fig. 6C), with no significant effects on body weight of the xenograft mice (Fig. 6B). It was also confirmed by immunohistochemistry that the injected LM1 cells were responsible for the origination of tumor and the metastasized tumor nodules in lung (Fig. 6D). In contrast, histological properties of major organs (Fig. 6E) and complete blood count (CBC) result (Fig. 6F) of the compound-administered mice remained normal. Thus, both ABA and APC appear to be promising antimetastatic agents that are unlikely to cause adverse effects in normal tissues.

## DISCUSSION

IDPs/IDPRs are important not only for normal cellular processes but also for the development of various human diseases. In particular, proteins validated as potential drug targets have been increasingly identified to contain IDPRs crucial for PPI mediation. However, the dynamic structure of IDPs/IDPRs limits their use in rational structure-based drug discovery. There are some successful examples of finding of compounds that can bind to and regulate the IDPR-containing proteins (e.g., the c-Myc IDPR-targeting compound 10058-F4). However, most of the current approaches to discover compounds targeting functional IDPR are based on random screening. Meanwhile, because many IDPRs undergo characteristic DOTs upon specific PPIs (*9*, *10*), related structural information can be retrieved from their complexed structures. This, together with the in-depth insights into the compound binding modes (*38*) and the rapidly accumulating knowledge of the IDPR structural properties (*6*, *7*), suggests the possibility for utilization of the structure-based rational approach as a feasible route for efficient discovery of drug leads targeting specific IDPRs engaged in DOT-based PPIs.

The present novel approach to an antimetastatic agent development provides a prime example of a collaborative work of in silico, in vitro, in cell, and in vivo analyses to discover the drug candidates targeting a pharmacologically important IDPR. In particular, we propose here a three-step computational platform for finding these drug leads. First, IDPRs with DOT potential are selected as potential drug-target sites. We speculate that these regions can be identified based on the characteristic features of their intrinsic disorder predisposition profiles similar to those observed in the known DOT-based PPI regions of MBD2 (residues 360 to 393) and c-Myc (residues 395 to 430) (Fig. 1B). Second, for virtual screening, ordered conformation is taken from the structure of selected IDPR complexed with binding partner. Third, MD simulation is conducted for the selected drug leads targeting IDPRs. Because the structure of target IDPR is dynamic (*6*, *7*) and because the presumably entropy-driven compound binding also occurs in a dynamic fashion (*38*), MD simulations of the compound-target complex structures are essential for detailed evaluation of the binding feasibility. In this study, MD simulation indicated the compound binding–specific conformational perturbations of MBD2, particularly at its critical PPI site with p66α, which could provide a structural basis for the molecular inhibition of the DOT-based PPI of MBD2. In general, specific molecular interactions of IDPs/IDPRs are known to be accomplished in distinctive ways such as DOT, avidity, allovalency, and fuzzy binding; the last three involves multivalent binding sites, whereas the first represents a simple two-state binding involving a single binding site (*41*, *42*). The present MD simulation result suggests that the ABA and APC binding of the MBD2 IDPR resembled a dynamic, multivalent interaction at low entropic cost, rather than the DOT-based interaction relevant to its p66α binding. The entropy-driven compound binding and structural multiplicity of the compound-bound IDPR have been identified earlier in the case of 10058-F4 binding to c-Myc_402–412_, which also requires just a few stable atomic interactions (*38*, *39*). In this regard, increased fuzziness of the MBD2 IDPR by the compound binding may conversely lead to decreased propensity for DOT for its p66α interaction, although the exact mode of binding of our compounds to the MBD2 IDPR, which can ultimately underlie their PPI inhibition mechanism, remains to be characterized in detail.

Our computational platform also contains an additional in silico study using the SEA, which was practical to assess off-target probability of the suggested compounds that is potentially associated with adverse effects in actual usage. In subsequent studies, mRNA-Seq results in cells (Fig. 5J) were consistent with the SEA result (Fig. 2A) that predicted no significant off-target probability, and in vivo administration of the suggested compounds raised no significant toxicity in normal tissues (Fig. 6, E and F).

It is generally appreciated that identifying and understanding molecular regulation and signaling network involved in the EMT process are essential to provide a molecular basis for antimetastatic drug development (*43*, *44*). Concerning this study, we have recently identified the MBD2-p66α molecular system in Mi-2/NuRD CRC as a promising target for EMT modulation by observing the induction of MET (conversed process of EMT) by knockdown of MBD2 and/or p66α in cancer cells. Together with this parallel effort, the present discovery of novel antimetastatic agents targeting a component of Mi-2/NuRD CRC validates that this epigenetic machinery can serve as an emerging target system for efficient antimetastatic drug developments. Both ABA and APC disrupting the specific PPI of MBD2 were able to suppress cellular EMT processes, thereby inducing epithelial differentiation of the more aggressive CSCs. Last, our compounds potently inhibited the cancer metastasis in vivo. Furthermore, considering that they raised no noticeable adverse effects on blood and normal tissues, the present results provide a basis for a novel safe control of cancer metastasis. Hence, found in this study, low–molecular weight (<250 g mol^−1^) compounds constitute a pioneering example of antimetastatic agents acting on a specific Mi-2/NuRD CRC component. In addition, the present observation that the compound treatments rendered the cancer cells more sensitive to anticancer drugs (Fig. 5I) provides important implications in combination therapy for cancer.

In conclusion, this study successfully used a rational approach to search for the novel antimetastatic agents acting via inhibition of the DOT-based PPI in an IDPR. As IDPs/IDPRs play crucial roles in diverse cellular processes (*6*, *7*), we believe that this platform can be applied for the discovery of innovative drug leads targeting DOT-based PPI regions in proteins associated with various cancers and other diseases.

## MATERIALS AND METHODS

### Study design

This study was designed to develop a novel platform for the discovery of drug leads based on molecular docking and MD simulations of the DOT-associated IDPRs of target proteins and, as a proof of concept, to identify candidate drugs, suppressing metastatic potentials of cancer cells in vitro and in vivo, by targeting an IDPR of MBD2 that undergoes a DOT upon association with its binding partner p66α for the integration of the Mi-2/NuRD CRC. These objectives were addressed by (i) analyzing intrinsic disorder predispositions of drug-target proteins and evaluating potential disorder-based binding regions (*45*), (ii) doing molecular docking with druggable compounds from the ZINC compound library to the potential drug-target sites, (iii) selecting two lead compounds based on the docking scores and off-target probabilities and experimental validation of target binding, (iv) evaluating the mode and efficiency of the compound binding via MD simulations, (v) assessing the identified leads for biological effects suppressing metastatic potentials of cancer cells, and (vi) verifying antimetastatic efficacy in a murine xenograft tumor model.

In animal studies, mice were randomly assigned to treatment and control groups. Numbers of tested mice were specified in each figure. Outliers were removed only if mice died at an early stage of the treatment according to the Hanyang University Institutional Animal Care and Use Committee (IACUC) dimension guideline. The primary end points were tumor size and cancer metastasis to lung. Mice were euthanized when moribund or at the end of the prespecified treatment period. All procedures were performed in accordance with institutional protocols approved by the IACUC of the Hanyang University. Pathology analysis was performed in a blinded fashion.

### Statistical analysis

Data were presented as means ± SE. The sample size for each experiment, *n*, was included in Results and the associated figure legend. Everywhere in the text, the difference between two subsets of data was considered statistically significant if the one-tailed Student’s *t* test gave a significance level *P* (*P* value) less than 0.05. Multiple comparisons, more than two means, were performed using a univariate analysis of variance (ANOVA), where a Scheffe posttest was performed in some cases or Kruskal-Wallis test. GraphPad Prism was used to generate MI_50_ curves for cell lines treated with ABA and APC in vitro. In addition, IC_50_ curves for FRET assay were also generated by GraphPad Prism. Statistical analyses were performed using IBM SPSS statistics 23.

## Supplementary Material

http://advances.sciencemag.org/cgi/content/full/5/11/eaav9810/DC1

Download PDF

Original data file S1

Rational discovery of antimetastatic agents targeting the intrinsically disordered region of MBD2

## SUPPLEMENTARY MATERIALS

Supplementary material for this article is available at http://advances.sciencemag.org/cgi/content/full/5/11/eaav9810/DC1 Supplementary Materials and Methods Fig. S1. Structural information on MBD2 and c-Myc. Fig. S2. SEA and cell migration analysis for the nine selected hit compounds targeting MBD2. Fig. S3. MD simulations of the selected compound-docked structures of MBD2 and c-Myc. Fig. S4. FRET dynamics of ABA and APC to the MBD2-p66α interaction. Fig. S5. Effects of ABA and APC on the expression of EMT markers and CSC properties in various breast and colon cancer cells. Table S1. Molecular docking result (H-bond, hydrogen bond; N/A, not available). Table S2. Selection of compound by in silico assessment of off-target probability by SEA analysis. Table S3. Backbone torsion angle variations (95% confidence interval) of the four key residues in the four different MD simulations of MBD2. Table S4. *T* test and *P* values on the backbone torsion angle summarized in table S3. Table S5. Primer sets for vector construction. Original data file S1. Figure 1D PDB files. References (*46*–*69*)

## Appendix

View/request a protocol for this paper from *Bio-protocol*.
