# Supplementary material for: Rational discovery of antimetastatic agents targeting the intrinsically disordered region of MBD2
Source: Sci Adv. 2019 Nov 20;5(11):eaav9810. doi: 10.1126/sciadv.aav9810 (PMC6867884; doi:10.1126/sciadv.aav9810)
Supplement: http://advances.sciencemag.org/cgi/content/full/5/11/eaav9810/DC1 [file supp_5_11_eaav9810__index.html]

Science Advances | Science AdvancesAAASSearchScience AdvancesMenu

## Supplementary Materials

**The PDFset includes:**

- Supplementary Materials and Methods
- Fig. S1. Structural information on MBD2 and c-Myc.
- Fig. S2. SEA and cell migration analysis for the nine selected hit compounds targeting MBD2.
- Fig. S3. MD simulations of the selected compound-docked structures of MBD2 and c-Myc.
- Fig. S4. FRET dynamics of ABA and APC to the MBD2-p66α interaction.
- Fig. S5. Effects of ABA and APC on the expression of EMT markers and CSC properties in various breast and colon cancer cells.
- Table S1. Molecular docking result (H-bond, hydrogen bond; N/A, not available).
- Table S2. Selection of compound by in silico assessment of off-target probability by SEA analysis.
- Table S3. Backbone torsion angle variations (95% confidence interval) of the four key residues in the four different MD simulations of MBD2.
- Table S4. *T* test and *P* values on the backbone torsion angle summarized in table S3.
- Table S5. Primer sets for vector construction.
- References (*46*–*69*)

Download PDF

**Other Supplementary Material for this manuscript includes the following:**

- Original data file S1 (.zip format). Figure 1D PDB files.

**Files in this Data Supplement:**

- Adobe PDF - aav9810\_SM.pdf
