# Supplementary material for: Rational discovery of antimetastatic agents targeting the intrinsically disordered region of MBD2
Source: Sci Adv. 2019 Nov 20;5(11):eaav9810. doi: 10.1126/sciadv.aav9810 (PMC6867884; doi:10.1126/sciadv.aav9810)
Supplement: Download PDF [file aav9810_SM.pdf]

Science Advances
AAAS

advances.sciencemag.org/cgi/content/full/5/11/eaav9810/DC1

Supplementary Materials for

Rational discovery of antimetastatic agents targeting the intrinsically disordered region of MBD2

Min Young Kim, Insung Na, Ji Sook Kim, Seung Han Son, Sungwoo Choi, Seol Eui Lee, Ji-Hun Kim, Kiseok Jang, Gil Alterovitz, Yu Chen, Arjan van der Vaart, Hyung-Sik Won*, Vladimir N. Uversky*, Chul Geun Kim*

*Corresponding author. Email: cgkim@hanyang.ac.kr (C.G.K.); wonhs@kku.ac.kr (H.-S.W.); vuversky@health.usf.edu (V.N.U.)

Published 20 November 2019, *Sci. Adv.* **5**, eaav9810 (2019)
DOI: 10.1126/sciadv.aav9810

The PDF file includes:

Supplementary Materials and Methods
Fig. S1. Structural information on MBD2 and c-Myc.
Fig. S2. SEA and cell migration analysis for the nine selected hit compounds targeting MBD2.
Fig. S3. MD simulations of the selected compound-docked structures of MBD2 and c-Myc.
Fig. S4. FRET dynamics of ABA and APC to the MBD2-p66α interaction.
Fig. S5. Effects of ABA and APC on the expression of EMT markers and CSC properties in various breast and colon cancer cells.
Table S1. Molecular docking result (H-bond, hydrogen bond; N/A, not available).
Table S2. Selection of compound by in silico assessment of off-target probability by SEA analysis.
Table S3. Backbone torsion angle variations (95% confidence interval) of the four key residues in the four different MD simulations of MBD2.
Table S4. *T* test and *P* values on the backbone torsion angle summarized in table S3.
Table S5. Primer sets for vector construction.
References (*46–69*)

Other Supplementary Material for this manuscript includes the following:

(available at advances.sciencemag.org/cgi/content/full/5/11/eaav9810/DC1)

Original data file S1 (.zip format). Figure 1D PDB files.

Supplementary Materials and Methods

Computational analysis to yield potential ligands

Protein sequence alignments were performed using Clustal O 1.2.1 (*46*). The protein structures retrieved from PDB (Protein Data Bank IDs 2L2L and 1NKP for MBD2 and c-Myc, respectively) were illustrated and inspected using PyMOL 1.3 (*47*). Intrinsic disorder propensities were predicted from the protein amino acid sequences, using the PONDR-FIT (*48*), VLXT (*49*), and VSL2 (*50*) algorithms. For molecular docking, structures of the $MBD2_{360-393}$ and $c\text{-}Myc_{395-430}$ were taken from Protein Data Bank IDs 2L2L and 1NKP, respectively. Molecular docking experiments to search for the potential ligands of MBD2 from the ZINC library of compounds (*13*) were performed using the DOCK 3.5.54 software (*51, 52*) with the 'startdockblaster5c' algorithm. In addition to using other default parameters, the 'vdW scale' was adjusted to 1.2, as its default value of 1.0 was not successful in generating meaningful docking positions. For the resulting 1,000 compounds that appeared to dock into MBD2, the DOCK binding scores and number of intermolecular hydrogen bonds were analysed using PyMol 1.3 to find compounds capable of the relatively more favorable complex formations.

To assess off-target probability of the hit compounds derived from the ZINC library screening, the web-based similarity ensemble approach (*14*) application (http://sea.bkslab.org) was utilized following its user instruction. Briefly, SEA evaluated target protein similarity based on the structural similarity between their ligand sets (*15, 16*). Running SEA search for our compounds explored total 2,060 of human proteins in the database and yielded Tanimoto coefficients (Tc) scores of the chemical similarity calculated for each pair of ligand sets. In addition, the E-values were derived from a statistical model to inversely indicate the stronger relation of two proteins. Finally, the combination of the Max Tc value over 0.5 and the E-value below 10^{-10} was considered as a significantly probable binding (*15*).

Molecular dynamics (MD) simulations

MD simulation parameters for the ABA and APC compounds were obtained using the CGenFF 0.9.7.1 beta program (*53*), embedded in the ParamChem web interface (*54*). Parameters for 10058-F4 were adjusted in comparison with the structural components of 'BCL6 inhibitor 57 ring system A' of the CHARMM 36 CGenFF force field (*36*). CHARMM parameters were converted into GROMACS format using the 'cgenff_charmm2gromacs.py' Python script from the MacKerell's Lab homepage (http://mackerell.umaryland.edu/charmm_ff.shtml). Starting configurations for MD simulations were prepared with the 'startdockblaster4c' algorithm of DOCK, under the same setup condition as in the previously described at 'startdockblaster5c' protocol. All MD simulations were performed using the GROMACS 5.0.4 software package (*55*) with the FFTW 3.3.4 library (*56*) in double precision, by applying the CHARMM 36 force field (*57*). A truncated octahedron box with a buffering area thickness of 1.2 nm was used, and periodic boundary conditions were in effect. The systems were solvated and neutralized in a 100 mM KCl solution of TIP3P water (*58*). After solvation, energy minimization using the steepest descent method was performed until the maximum force was less than 1,000 kJ mol^{-1} nm^{-1}, using an initial step size of 0.01 nm. During the heating process, short restrained MD simulations were performed in the NVT ensemble at three different temperatures: 100 K, 200 K, and 310 K. Initial velocities were generated according to the Maxwell distribution. Positional restraints with a force constant of 1,000 kJ mol^{-1} nm^{-2} were applied to all heavy atoms, and V-rescale was used to control temperature (*59*).

Equilibration at 310 K was performed in the NPT ensemble using three subsequent 1 ns positional restrained MD simulations with force constants of 1,000, 100, and 10 kJ mol^{-1} nm^{-2}. The pressure of 1 bar was controlled using the Parrinello-Rahman barostat with an

isothermal compressibility of $4.5 \cdot 10^{-5}$ bar^{-1} (*60*, *61*). Equilibration was followed by 50 ns NPT production runs in the absence of positional restraints. Coordinates were stored every 10 ps for further analysis. All simulations used the LINCS method (*62*) to constrain bonds with H-atoms, the Verlet cut-off scheme to update the neighbor list every 10 fs, a cut-off of 1.2 nm for the electrostatics and van der Waals interactions, the particle mesh Ewald method (PME) to treat long-range electrostatics (*63*, *64*), and a time-step of 2 fs.

Backbone root-mean square deviations (RMSDs) were calculated using GROMACS 5.0.4 ‘rmsdist’ with the energy minimized structure for reference. Residue backbone root-mean square fluctuations (RMSFs) were measured using GROMACS 5.0.4 ‘rmsf’. Contact numbers were calculated using GROMACS 5.0.4 ‘mindist’, using a distance criterion of 3 Å between any atom of the protein and compound. Another cut-off 3.6 Å between heavy atoms (C, N, O, S) was utilized to evaluate the MBD2-APC and p66α-ABA binding. The application of two criteria (3 Å and 3.6 Å) was to double-check the binding status. Extra attention was given to D_{368} contacts within 3.6 Å cutoff. Interaction energies were calculated using the ‘NAMDenergy’ plugin embedded in VMD 1.9.1 (*65*, *66*). φ and ψ backbone dihedral angles were calculated using GROMACS 5.0.4 ‘rama’. R 3.1.2 was applied to calculate T test *p*-values.

Plasmid construction

For prokaryotic expression and purification of His-tagged MBD2 and truncated $p66\alpha^{1-206}$proteins, PCR products of full length MDB2 cDNA or p66α cDNA fragment was amplified from pDsRED2-C1-MBD2 (donation from Dr. Gerd P. Pfeifer, Beckman Research Institute of City of Hope, USA) and pEF1α-3XFB-p66α vector, respectively. The MBD2 and $p66\alpha^{1-206}$ PCR products were cloned into the pRSET-eYFP-cas-dTomato (donation from Dr.

Young Pil Kim, Hanyang University, Korea) expression vector using *BamH*I and *EcoR*I, or *Kpn*I and *Hind*III, respectively. Primer sequences for PCR reaction used were listed in Supplementary Table S5. For FRET assay in cell, the Fluorescence dye-tagged full-length p66α and MBD2 plasmids (mcherry-p66α-pcDNA3 and peYFP-N1-MBD2) were constructed using mCherry-hCdt1(1/100)Cy(-)/pcDNA3 (RIKEN BRC, RDB15459) and peYFP-N1 vectors, respectively. As the first step for generating the fluorescence dye tagged construct, PCR products of full length p66α and MBD2 cDNAs were amplified from pEF1α-3XFB p66α (*33*) and pDsRED2-C1-MBD2 (donation from Dr. Gerd P. Pfeifer, Beckman Research Institute of City of Hope, USA). The PCR product of the full-length p66α cDNA was inserted into the *Xho*I- and *Xba*I-digested mCherry-pcDNA3 expression vector. In addition, the eYFP cDNA was amplified by PCR using the pRSET-YFP-cas-dTomato vector as a template and cloned into the *Age*I- and *Not*I-digested pEGFP-N1 expression vector. The PCR product of full-length MBD2 cDNA was subcloned into peYFP-N1 expression vector using *Bgl*II and *EcoR*I. The integrity and identity of all constructs were confirmed by DNA sequencing.

Purification of recombinant bacterial proteins

His-dTomato-MBD2 and His-eYFP-p66$\alpha^{1\text{-}206}$ were expressed in BL21(DE3)pLysS bacteria (Promega, L1195) and purified as described elsewhere (*67*). Bacteria were grown in LB-amp until OD 600 = 0.4~0.6. After which, 0.4 mM IPTG was applied for 2 h at 37°C to induce protein expression. Cultures were centrifuged at 5,000 rpm for 15 min at 4°C and lysed in 1×10^7 cell/25 μL lysis buffer (50 mM NaH_2PO_4, 300 mM NaCl, 10 mM Imidazole, and 1 mM PMSF) for 30 min, sonicated 2 times with 4 pulses, and placed on ice 10 second. After adding Triton-X (1% final), lysates were centrifuged at 12,000 rpm for 20 min at 4°C. Supernatants were applied to 500 μL bed volume of Ni-NTA agarose (Qiagen, 30210) and rotated at 4°C overnight. Beads were washed with 1 mL washing buffer (50 mM NaH_2PO_4,

300 mM NaCl, 20 mM Imidazole, and 1 mM PMSF), and His-fusion proteins were eluted with elution buffer (50 mM NaH_2PO_4, 300 mM NaCl, 250 mM Imidazole, and 1 mM PMSF).

Cell culture and chemical materials

The human lung metastatic breast cancer (the LM1 line of MDA-MB-231 and its' GFP-tagged derivative; donation from Prof. Su-Jae Lee, Hanyang University, Korea), human luminal ER+ breast cancer (MCF7, ATCC no. HTB22) and human embryonic kidney (293T, ATCC no. CRL-3216) cell lines were maintained in Dulbecco's modified Eagle's medium (DMEM; HyClone, SH30243.01) supplemented with 10% fetal bovine serum (FBS; HyClone, SH30084.03). The human triple negative breast cancer (HCC1806, ATCC no. CRL-2335; MDA-MB-468, ATCC no. HTB-132), human luminal ER+ breast cancer (T47D, ATCC no. HTB133), and human colon cancer (HCT116, ATCC no. CCL-247; HT29, ATCC no. HTB38) cell lines were cultured in RPMI 1640 (HyClone, SH30027.01) supplemented with 10% FBS (HyClone, SH30084.03). The human luminal ER+, HER2+ breast cancer (BT474, ATCC no. HTB20) cell line was maintained in DMEM/F-12 (HyClone, SH30027.01) supplemented with 10% FBS (HyClone, SH30084.03). The non-tumorigenic human breast epithelial (MCF10A, ATCC no. CRL-10317) cell line was maintained in DMEM/F-12 (HyClone, SH30027.01) supplemented with 5% horse serum (Gibco, 16050-122), 20 $ng \cdot mL^{-1}$ epidermal growth factor (EGF; Peprotech, 16050-122), 10 $\mu g \cdot mL^{-1}$ insulin (Sigma Aldrich, I1882), 0.5 $\mu g \cdot mL^{-1}$ hydrocortisone (Sigma Aldrich, H4001) without cholera toxin. All cell lines were added 1% penicillin/streptomycin and cultured in a 5% CO_2 atmosphere. The chemical compounds 2-amino-N-(2,3-dihydro-benzo[1,4]dioxin-2-ylmethyl)-acetamide (ABA), 3-(2-amino-acetylamino)-pyrrolidine-1-carboxylic acid tert-butyl ester (APC), and chemical compounds #4 and #7 were purchased from Fluorochem (UK). Chemical compounds #5, #9 and #10 were purchased from Ambinter (USA), whereas

chemical compound #6 was purchased from Enamine (USA). All of compounds was prepared as 1 M stock solutions in dimethyl sulfoxide (DMSO).

RNA preparation for sequencing

Total cellular RNA was extracted using Qiazol reagent. The RNA quality was examined by spectrophotometry, agarose gel electrophoresis (calculating the 18S and 28S rRNA ratio) and an Agilent Technologies 2100 Bioanalyzer (ensuring a RIN value greater than 7). The library was prepared using the QuantSeq 3' mRNA-Seq Library Prep Kit FWD. The constructed libraries were subjected to 75-bp single-end sequencing using an Illumina NextSeq 500 sequencer at the Ebiogen. All procedures were performed following the manufacturer's instructions.

FRET *in vitro* and in cell

For the *in vitro* FRET assay, the donor (eYFP-p66$\alpha^{1\text{-}206}$) and acceptor (dTomato-MBD2) proteins were purified from the IPTG-induced BL21(DE3)pLysS (Promega, L1195) strain of *E.coli*. Various concentrations of ABA and APC were mixed with 1 μM of each purified donor-acceptor proteins in binding buffer (100 mM KCl, 10 mM Tris–HCl, pH 7.9, 1 mM EDTA, 1 mM DTT, 4% glycerol) and incubated at 37°C for 30 min. The reaction mixtures were seed on 96-well plate. Emission spectra from 500 to 600 nm, upon excitation at 480 nm with a bandwidth 2 nm, were obtained in a Varioskan Flash Spectral Scanning Multimode Reader (Thermo Scientific). The mean FRET efficiency (R_{FRET}) was calculated as $R_{FRET} = I_A/(I_A + I_D)$, where I_A and I_D represent acceptor and donor intensities, respectively. For FRET assay in the cells, 293T cells were grown on glass-bottom dishes (SPL Life Sciences, 200350) in DMEM supplemented with 10% FBS (HyClone,

SH30084.03), then transfected with plasmids (mCherry-p66α and eYFP-MBD2) using Effectene (Qiagen, 301425). One day after transfection, various concentrations of ABA or APC were added into the cells for 24 h, and then culture medium was replaced with DMEM without phenol red (Welgene, LM001-10). FRET imaging was conducted with a confocal microscope (Nikon C2si). For the in-cell FRET analysis, the donor fluorescence was excited at 488 nm by a laser, and emission of the acceptor was collected through a 570/613 nm filter (Nikon, 67-006-NKN). Excitation and emission for eYFP fluorescence were 488 nm and 525/561 nm, whereas those were 550 nm and 570/613 nm for mCherry fluorescence, respectively. For the quantification of FRET efficiency, emission value at 588 nm, upon excitation at 488 nm, were obtained in a Varioskan Flash Spectral Scanning Multimode Reader (Thermo Scientific). Image processing was performed using the ImageJ (ver. 1.51) software. The relative FRET ratios for compounds were calculated by $FRET_{comp}/FRET_{mock}$.

Immunoblotting

Cultured cells were harvested in PBS and resuspended in ice-cold lysis buffer (50 mM Tris-HCl, pH 7.4, 150 mM NaCl, 1 mM EDTA, 1% NP-40, and 1 mM PMSF). Aliquots of the whole cell extract proteins were electrophoresed on 10% SDS-PAGE and transferred to PVDF membranes (GE Healthcare, 10600069). Membranes were cut by two in the middle (~50 KDa based on the protein marker), and the individual membranes were subjected to immunoblotting with different antibodies. After blocking with PBS containing 5% non-fat dry milk in a solution of 0.1% Tween 20, membranes were incubated with the following antibodies: SNAIL (Cell Signaling, 3879, 1:2000), CTNNB1 (Santa Cruz, sc-7199, 1:1500), CDH1 (Santa Cruz, sc-8426, 1:1500), CDH2 (Santa Cruz, sc-7939, 1:1500), SLUG (Santa Cruz, sc-10436, 1:1500), ZEB1 (Santa Cruz, sc-10572, 1:2000), TWIST1 (Santa Cruz, sc-

15393, 1:1500), MBD2 (Abcam, ab38646, 1:2500), and p66α (Abcam, ab87663, 1:2500). The blots were incubated with their respective HRP conjugated secondary antibodies at room temperature for 1 h. The HRP conjugated secondary antibodies used in immunoblot were as follows: anti-mouse IgG HRP (Thermo, 31430, 1:10000), anti-goat IgG HRP (Thermo, 811620, 1:10000), anti-rabbit IgG HRP (Abcam, ab6802, 1:10000). Polyclonal anti-ACTB antibody (Santa Cruz, sc-1616, 1:2500) was used as the loading control for immunoblotting. Proteins were visualized by chemiluminescence using an ECL system (GE Healthcare, RPN2106). Relative amounts of proteins in several cell lines were quantified using ImageJ (ver. 1.51).

Co-immunoprecipitation (Co-IP) assay

Co-IP experiments were performed as described previously (*67*). Various concentration of ABA or APC were treated into LM1 cells. Two days after treat, cell lysates were harvested in lysis buffer (50 mM Tris–HCl, pH 7.4, 150 mM NaCl, 1 mM EDTA, 1% NP-40 and 1 mM PMSF) with freshly added 1 mM DTT and protease inhibitor cocktail (Sigma Aldrich, P8340). Input samples (10% of IP protein) were saved for immunoblot analysis. For immunoprecipitation, equal amounts of lysate protein (1~2 mg, determined by Bradford protein assay) were incubated with 1 μg of appropriate anti-MBD2 (Abcam, ab38646) and anti-p66α (Abcam, ab87663) antibodies and protein A/G agarose beads (Pierce, 20421). Beads were then eluted with 2× bed volume of 0.2 M glycine buffer, followed by neutralization with an equal volume of 1 M Tris-HCl (pH 8.0). Precipitated proteins were separated by 10% SDS-PAGE and transferred to PVDF membranes (GE Healthcare, 10600069). Antibodies against MBD2 (1:2500) and p66α (1:2500) were used for immunoblotting.

Immunocytochemistry

Cells grown on each coverslip were pre-treated with or without ABA and APC (10 μM) for 48 h. Cells were fixed with acetone and then probed at room temperature for 2 h with primary antibody against VIM (Santa cruz, sc-53464) or CDH1 (Santa cruz, sc-8426) diluted 1:250 in PBS. After washing with PBS, immunoreactivity was detected with Cy3-labeled goat anti-mouse IgG (Thermo Fisher, M30010), and then counterstained with DAPI for 5 min to detect nuclei. The images were visualized and obtained using a confocal microscope (Nikon C2si). Image processing was performed using the ImageJ (ver. 1.51) software.

Cell proliferation & Chemo-resistance assays

For the ABA and APC drug effect tests, a total of 3×10^3 cells·well^{-1} of cells were seeded onto 96-well plates in triplicate and pre-treated with or without ABA and APC (10 μM) for 48 h. Cell proliferation ability was measured using 3-(4,5-dimethylthiazol-2-yl)-2,5-diphenyltetrazolium bromide (MTT; Sigma-Aldrich, M2128) colorimetric assay. For chemo-resistance assay, cells were culture in the absence or presence of various concentration of doxorubicin (Sigma Aldrich, 44583) or cisplatin (Sigma Aldrich, P4394) for additional 48 h after ABA and APC pre-treatment, and the cell viability was measured using MTT colorimetric assay. All MTT solution (5 mg·mL^{-1}) was added to the cultures and the cells were incubated for additional 3 h at 37°C and 5% CO_2. MTT medium was discharged, and the formazan crystals were dissolved using 100 μL DMSO per well. The formazan concentration was measured spectrophotometrically at 490 nm using a Multiskan EX (Thermo Fisher, 51118170).

Sphere formation assays

A total of 1×10^2 cells·well^{-1} of cells were seeded in triplicate onto 96-well plates after coated with 10% poly (2-hydroxyethyl methacrylate) (polyHEMA) in serum-free DMEM/F12 with B27 supplement (Gibco, 17504-044), 20 ng·mL^{-1} basic fibroblast growth factor (bFGF; Thermo Fisher, 13256-029) and 20 ng·mL^{-1} EGF. For measure sphere formation activities, cells were treated with or without ABA and APC (10 μM) for 5 days, and the number of spheres (diameter > 50 μm) in each well was counted using an Olympus IX71 microscope with an Olympus DP72 camera.

Flow cytometry

For flow cytometric analysis of cell cycle, cells were treated with or without ABA and APC (10 μM) for 48 h, washed and suspended with pre-chilled PBS, and then fixed with pre-chilled 70% ethanol at -20°C. Fixed cells were incubated with 20 μg·mL^{-1} RNase A at 37°C for 30 min and stained with 50 μg·mL^{-1} propidium iodide (PI) at room temperature for 20 min. Samples were immediately analysed by FACS Canto II flow cytometer (BD Biosciences). The cell fractions in G1, S, and G2/M phases were quantified in histograms with FACSDiva software (BD Biosciences). For the identification of cell surface markers (CD44 and CD24), cells in PBS were stained fluorochrome-conjugated CD24-PE (BD Biosciences, 555428) and CD44-APC-Cy7 (Biolegend, 103028) antibodies for 15 min at room temperature in dark condition. Cells were sorted using FACS Canto II flow cytometer (BD Biosciences) and classified into four subsets.

Cell migration and invasion analysis

Transwell chambers were purchased from Corning Life Science (8 μm, 24-well insert; #3422). LM1 or HCT116 (1×10^4) cells in 0.1 mL of FBS-free medium containing various

concentrations of identified compounds were seeded into the upper chamber and incubated for 48 h. For invasion assays, 1×10^4 cells in 0.1 mL of FBS-free medium were seeded into the upper chamber of an 8 μM Matrigel (BD Biosciences, 354248)-coated chamber and incubated for 48 h. The lower chambers were filled with medium containing 10% FBS, and the same concentration of compounds as the upper chamber. Cells that had migrated and invaded through the Matrigel were fixed with methanol and then stained with Diff Quick Stain Kit (Sysmex Corporation, #38721). The whole membrane attached with migrated and invaded cells was photographed under 40× microscopic fields and the cell density in each membrane was measured by ImageJ for statistical analyses.

Wound healing analysis

Cells were seeded in 12-well plates and incubated until approximately 90-95% confluence. Wounds were generated by scraping the monolayer with a micropipette tip. Thereafter, the cells were cultured for 0 ~ 48 h under different experimental conditions: PBS-, ABA- or APC-containing medium. An Olympus IX71 microscope with an Olympus DP72 camera was used to image cells at the first time point (T_0) and the last time point ($T_{\text{end point}}$). For data analysis, ImageJ was used to measure the remaining wound area at T_0 and $T_{\text{end point}}$. The migration area was obtained by subtracting area at $T_{\text{end point}}$ from area at T_0.

Animal studies

The GFP-tagged LM1 cells were used for xenografts. Six-week-old immunodeficiency mouse (NPG mouse, Woojung Bio., Co. Ltd) were randomly divided into five groups (8 mice per group), followed by subcutaneous injection of single cell suspension (approximately 5×10^6 LM1 cells in 50 μL of PBS with 4 mg·mL-1 Matrigel) into mammary fat pads. 10 days after inoculation, mice were injected i.v. with ABA and APC (10 or 20 μg·kg-1) or PBS, a

total of 6 times, once every 3 days. Tumor volume was calculated using the formula (length × width^2)/2 every 3 days. The mice were sacrificed 4 days after the last injection and dissected for histological analysis. At the time of killing, original tumors were removed and weighted. The number of lung metastatic tumor nodule was counted in naked eyes after fixation in 10% formalin neutral buffer solution. For histological analysis, major organs were immediately fixed in 10% formalin neutral buffer solution, dehydrated in a graded series of ethanol, treated with xylene, paraffin-embedded, and cut into 4 μm sections. Hematoxylin and eosin (H&E)-stained tissue sections were analysed and photographed under the light microscope, and the representative histological images were recorded at ×400 magnification. Histopathological scoring for the metastatic tumors in the major organ was assessed by pathologist as suggested (*68*).

Immunohistochemistry (IHC)

Antigen retrieval of tissue sections was performed by autoclaving the samples for 30 min in 10 mM sodium citrate buffer (pH 6.0). Endogenous peroxidase activity was blocked by incubating sections with 3% hydrogen peroxidase solution for 15 min. Sections were incubated with rabbit polyclonal anti-GFP antibody (Novus, NB600-308) at 4°C overnight. Primary antibodies were diluted 1:200 using Dako antibody diluent solution (Dako, S0809). After washing twice in Tris buffer (pH 7.4), sections were serially incubated with post Primary and Novolink Polymer (Novolink Polymer Detection System, Leica, RE7150-K) for 30 min. Immunoreactivity was visualized by adding diaminobenzidine (*69*) substrate for 3 min followed by counterstaining of nuclei with Mayer’s hematoxylin.

SUPPLEMENTARY FIGURES

A

Protein	UniProt ID	PDB (chain)	PDB residues	UniProt residues	PDB binding residues	UniProt binding residues
MBD2	Q9UBB5	2L2L (B)	211-244	360-393	217-234	366-383
c-Myc	P01106	1NKP (A)	900-981	353-434	949-959	402-412

B

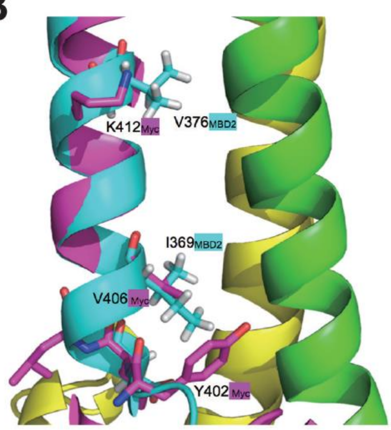

C

```
CLUSTAL O(1.2.1) multiple sequence alignment

MBD2:360-393    360 -----------KAFIVTDEDIRKQEERVQQVRKKLEEALMADILS 393
c-Myc:400-434   400 TAYILSVQAEEQKLISEEDLLRKRRE---QLKHKLEQL------- 434
                               : :*  :: :**:.*   *:::***:

CLUSTAL O(1.2.1) multiple sequence alignment

MBD2:366-383    366 -DEDIRKQEERVQQVRKKL 383
c-Myc:402-412   402 YILSVQAEEQK-------- 412
                       .:: :*::
```

Fig. S1. Structural Information on MBD2 and c-Myc. (**A**) Sequence information pertaining to the MBD2 and c-Myc regions used in computational analysis. (**B**) Structural comparison of MBD2 (sky blue; chain B, first frame of ensemble, PDB 2L2L) and c-Myc (magenta; PDB 1NKP) in their coiled-coil states complexed with cognate partners p66α (green; chain A; first frame of ensemble, PDB 2L2L) and Max (yellow; chain B, PDB 1NKP). Core sites of the intermolecular interactions of the complexes are emphasized by superimposition of the corresponding regions of MBD2 (residues 366-383) and c-Myc (402-412). Sidechains are presented for the ligand-targeted residues in the molecular docking experiments. (**C**) Pairwise sequence alignments of the selected regions of MBD2 and c-Myc, such as coiled-coil parts (residues 360-393 and 400-434 in MBD2 and c-Myc, respectively,

the regions used at structural alignment in panel B) and the ligand-binding sites (residues 366-383 and 402-412 in MBD2 and c-Myc, respectively).

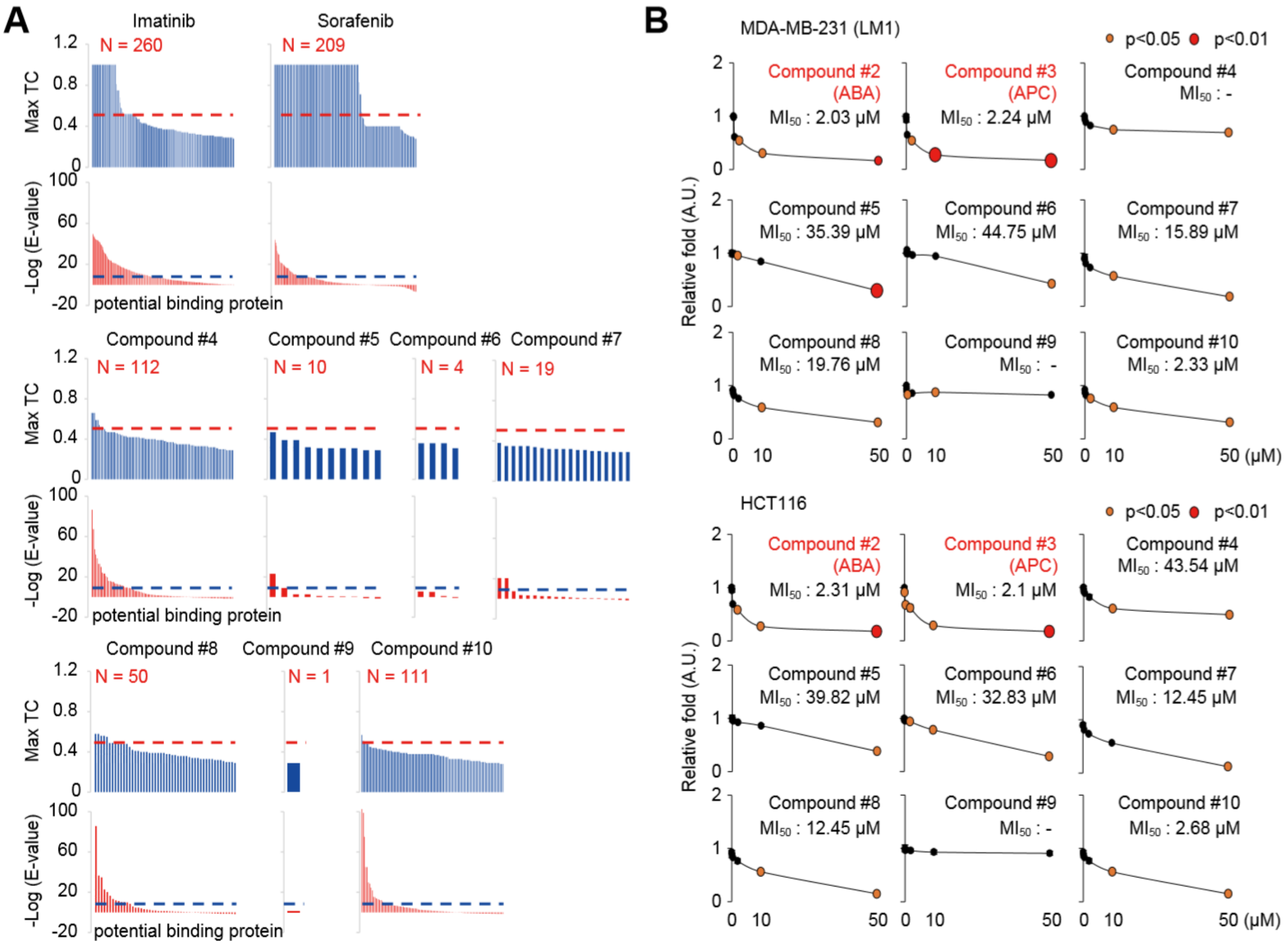

Fig. S2. SEA and cell migration analysis for the nine selected hit compounds targeting MBD2. (A) SEA analysis of hit compounds targeting MBD2. Max TC and E-value of the predicted binding are plotted for the N (number of potential targets predicted) off-target candidates. Two known anticancer drugs, imatinib (Gleevec®) and sorafenib (Nexavar®), were compared as control experiments. See Fig. 2A for 10058-F4 (additional control) and the two selected leads (compounds #2 and #3). (**B**) Cell migration assay results for the 9 selected hit compounds. The LM1 and HCT116 cells were fixed and stained after 48 h of Transwell migration in the absence and presence of individual compounds. Relative fold of the number of migrated cells counted are plotted over the compound concentration to yield MI_{50} value. Data (means ± SD, $n = 2$) were analyzed using Student's *t*-test. $^{**}P < 0.01$ and $^{*}P < 0.05$.

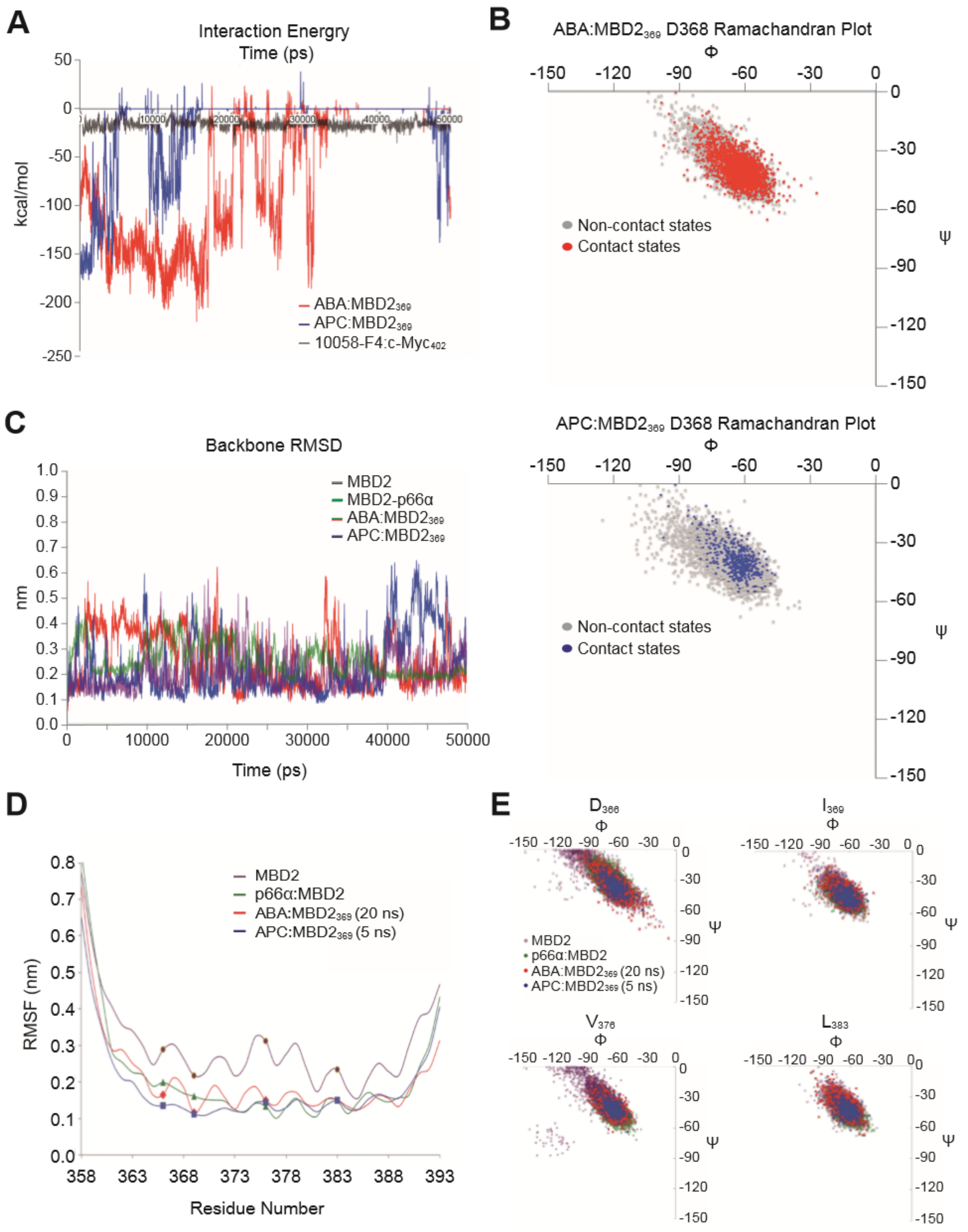

Fig. S3. MD simulations of the selected compound-docked structures of MBD2 and c-Myc. (**A**) Fluctuations of the compound-protein interaction energies during 50 ns trajectories

of the three indicated MD simulations. (**B**) Ramachandran plots showing distributions of the backbone torsion angles (Φ and Ψ) for the D_{368} residue in the MD trajectories of the compound-docked MBD2 models (ABA:$MBD2_{369}$ and APC:$MBD2_{369}$). The individual torsion angles were extracted separately at the compound-contacting and non-contacting states. (**C**) Backbone root mean square deviations (RMSDs) of MBD2 during the MD simulations in compound-free, compound-bound (ABA:$MBD2_{369}$ and APC:$MBD2_{369}$), and p66α-bound states. (**D**, **E**) Inhibition of the DOT-entailing PPI of MBD2 by the lead compounds. (**D**) MD simulation results presented by root-mean-square fluctuations (RMSFs) of the MBD2 backbone atomic positions in the absence and presence of bound molecules. Dots on the plot represent the positions of key PPI residues of MBD2. (**E**) Ramachandran plots describing the backbone torsion angle variations of the four key PPI residues in individual MD simulations.

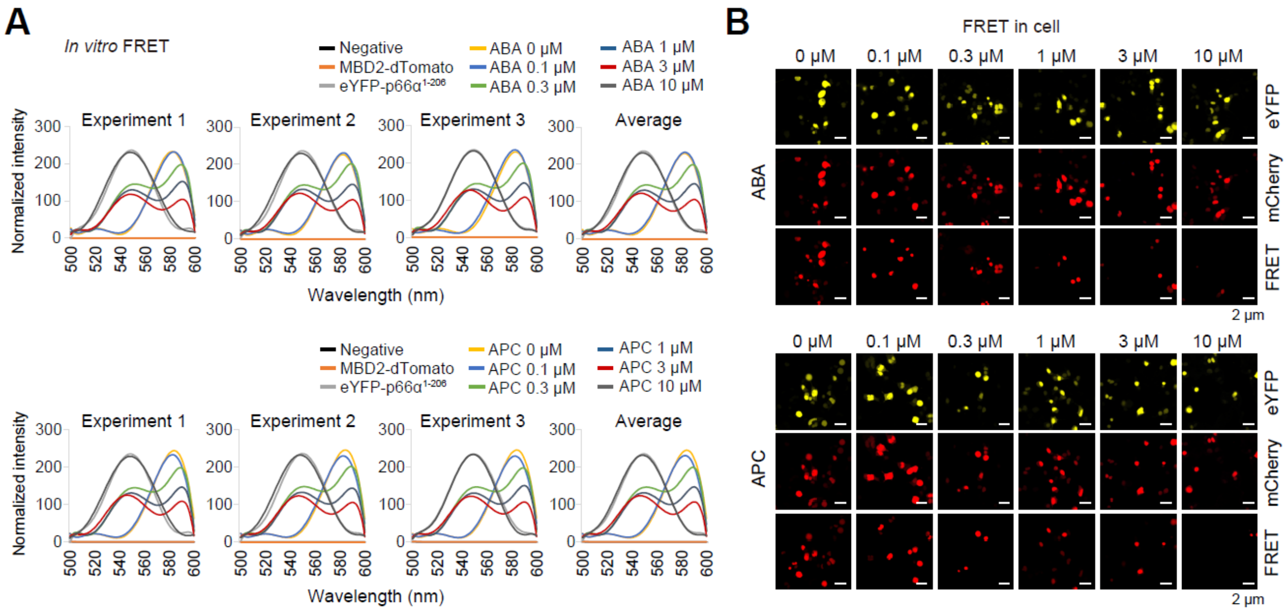

Fig. S4. **FRET dynamics of ABA and APC to the MBD2-p66α interaction.** (**A**) Graphs show the distribution of the *in vitro* FRET states of ABA (top) and APC (bottom). Fluorescence spectra were obtained from 500 nm to 600 nm (at an excitation wavelength of 480 nm). Intensities of each experimental sample were normalized to the averaged emission for dTomato stimulation in samples where fluorescent proteins were not included. Averaged FRET dynamics were obtained by 3 individual experiments. (**B**) Representative immunofluorescence microscopic photos of cells where various concentrations of ABA (top) or APC (bottom) treated. (Photo credit: S.H.S., Hanyang University).

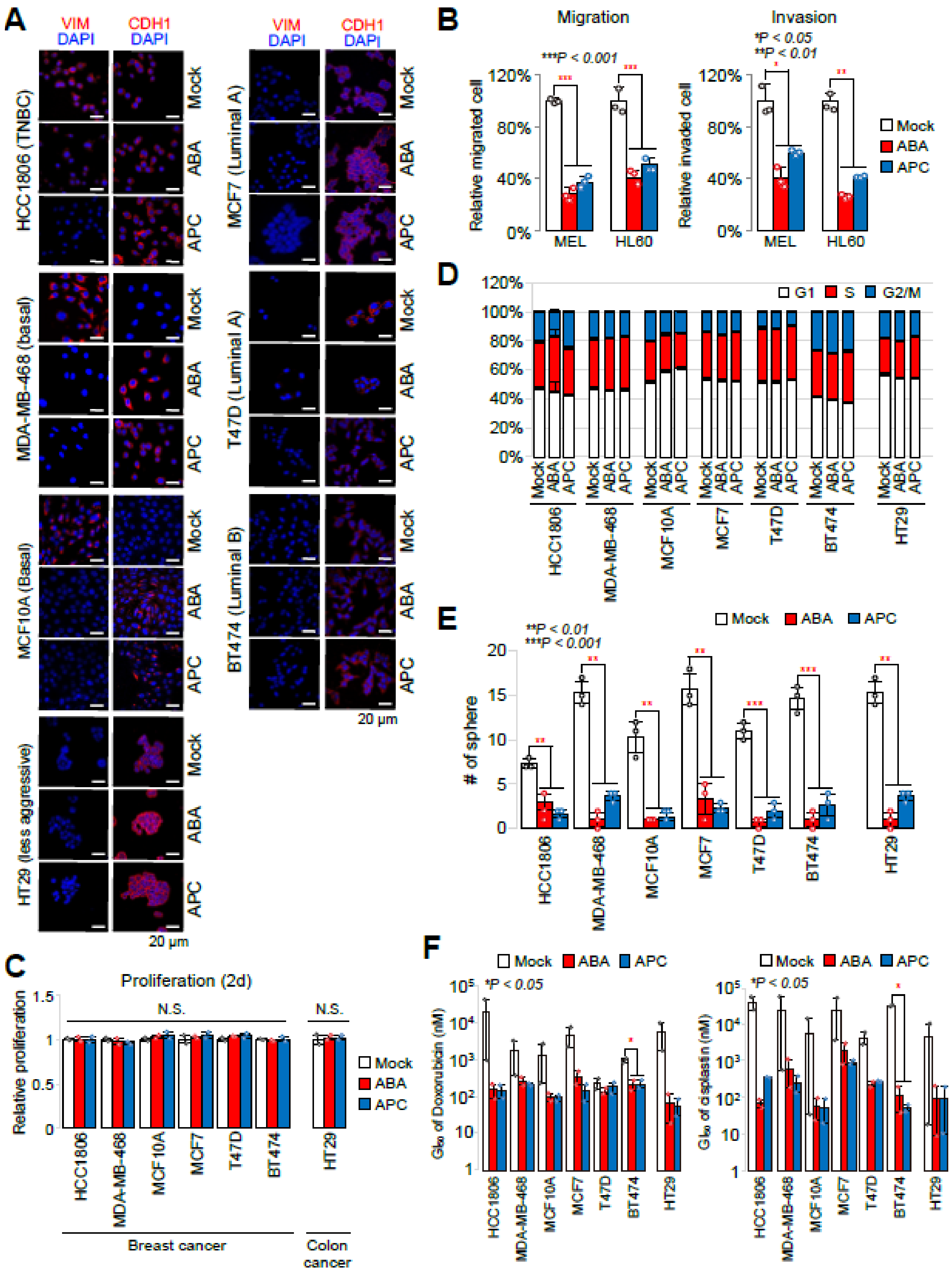
A
VIM
DAPI
CDH1
DAPI
HCC1806 (TNBC)
MDA-MB-468 (basal)
MCF10A (Basal)
HT29 (less aggressive)
MCF7 (Luminal A)
T47D (Luminal A)
BT474 (Luminal B)
Mock
ABA
APC
20 μm
B
Migration
Invasion
***P < 0.001
*P < 0.05
**P < 0.01
Relative migrated cell
Relative invaded cell
MEL
HL60
Mock
ABA
APC
C
Proliferation (2d)
Relative proliferation
N.S.
HCC1806
MDA-MB-468
MCF10A
MCF7
T47D
BT474
HT29
Breast cancer
Colon cancer
D
G1
S
G2/M
E
**P < 0.01
***P < 0.001
# of sphere
F
*P < 0.05
GI50 of Doxorubicin (nM)
GI50 of cisplastin (nM)

Fig. S5. Effects of ABA and APC on the expression of EMT markers and CSC properties in various breast and colon cancer cells. (**A**) Representative immunofluorescence images showing fluorescence signals for VIM or CDH1 (red), and DAPI (blue). (Photo credit: S.H.S., Hanyang University). (**B**) Migration (left) and invasion (right) abilities of the ABA or APC treated cells compared to parental cells. (**C**) Relative cell proliferation rates quantified by MTT assay after 2 days. $n = 2$. (**D**) Cell cycle analysis by FACS. $n = 2$. (**E**) Number of spheres counted by naked eye after 5 days. $n = 3$. (**F**) Chemosensitivity to doxorubicin (left) and cisplatin (right) quantified by MTT assay. $n = 2$. In all experiments, 10 μM of ABA or APC was treated. Data (means ± SD) were analyzed using Student's *t*-test. ***$P < 0.001$, **$P < 0.01$ and *$P < 0.05$.

SUPPLEMENTARY TABLES

Table S1. Molecular docking result (H-bond, hydrogen bond; N/A, not available).

A

#	Structure	ZINC ID	Energy Score (kcal/mol)	Number of H-bond	c-Myc residue contributions at H-bond	Net Charge (pH 7.0)	MW (g/mol) (pH 7.0)
1	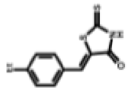	12406716 (10058-F4)	-6.77	1	c-Myc Y402 : 1	0	249.36

B

#	Structure	ZINC ID	Energy Score (kcal/mol)	Number of H-bond	MBD2 residue contributions at H-bond	Net Charge (pH 7.0)	MW (g/mol) (pH 7.0)
1	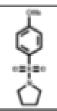	C00182069	-37.1	0	N/A	0	241.312
2	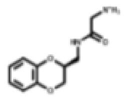	C40430779	-35.2	3	E_{373} : 2 Q_{377} : 1	1	223.252
3	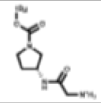	C60177071	-33.3	3	E_{373} : 2 Q_{377} : 1	1	244.315
4	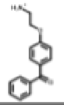	C05735453	-32.3	2	E_{373} : 1 Q_{377} : 1	1	242.298
5	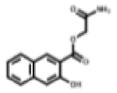	C03254317	-29.9	1	E_{373} : 1	0	245.234
6	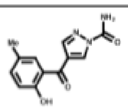	C14248970	-29.8	1	E_{373} : 1	0	245.238
7	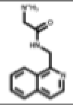	C79422185	-29.5	1	E_{373} : 1	0	216.214
8	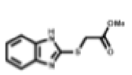	C00163241	-29.4	0	N/A	0	222.269
9	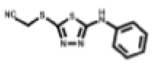	C03370619	-29.1	0	N/A	0	248.336
10	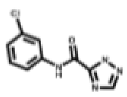	C05731012	-28.6	2	E_{373} : 2	0	222.635

A, 10058-F4 and c-Myc$_{396\text{-}430}$ molecular docking result. **B,** Top 10 compounds showing the most favourable binding to MBD2 in the molecular docking screening of ZINC compound library.

Table S2. Selection of compound by in silico assessment of off-target probability by SEA analysis.

	Number of protein			
Compound	Total	Max Tc > 0.5 (%)	E-Value < 1E-10 (%)	Max Tc > 0.5 and E- Value < 1E-10 (%)
Imatinib	260	80 (30.77)	93 (35.77)	35 (13.46)
Sorafenib	209	129 (61.72)	32 (15.41)	26 (12.44)
10058-F4	34	12 (35.29)	8 (23.53)	4 (11.76)
#1	114	17 (14.91)	35 (30.70)	10 (8.77)
# 2 (ABA)	8	1 (12.50)	0 (0.00)	0 (0.00)
# 3 (APC)	14	2 (14.29)	3 (21.43)	0 (0.00)
# 4	112	11 (9.82)	26 (23.21)	6 (5.36)
# 5	10	0 (0.00)	1 (0.91)	0 (0.00)
# 6	4	0 (0.00)	0 (0.00)	0 (0.00)
# 7	19	0 (0.00)	2 (10.53)	0 (0.00)
# 8	50	5 (10.00)	12 (24.00)	0 (0.00)
# 9	1	0 (0.00)	0 (0.00)	0 (0.00)
# 10	111	2 (1.80)	17 (15.32)	2 (1.80)

Protein binding affinity, as revealed by Similarity Ensemble Approach (SEA) analysis, is considered to be significant when Max Tc value > 0.5 and E-value < 1E-10.

Table S3. **Backbone torsion angle variations (95% confidence interval) of the four key residues in the four different MD simulations of MBD2.** For the ABA:$MBD2_{369}$ (20 ns period) and APC:$MBD2_{369}$ (5 ns period) MD simulations, the torsion angles were extracted at the compound-contacting statuses of the residues.

Residue	MD simulation	Φ	Ψ
D_{366}	ABA:$MBD2_{369}$	(-60.75,-59.91)	(-37.22,-36.38)
	APC:$MBD2_{369}$	(-61.49,-60.07)	(-36.82,-35.50)
	MBD2	(-74.67,-73.43)	(-23.32,-22.10)
	MBD2-p66α	(-63.59,-63.14)	(-32.15,-31.68)
I_{369}	ABA:$MBD2_{369}$	(-63.36,-62.76)	(-42.61,-42.04)
	APC:$MBD2_{369}$	(-63.53,-62.40)	(-43.59,-42.48)
	MBD2	(-64.72,-64.25)	(-41.26,-40.80)
	MBD2-p66α	(-63.27,-62.93)	(-44.61,-44.30)
V_{376}	ABA:$MBD2_{369}$	(-61.77,-61.24)	(-42.00,-41.44)
	APC:$MBD2_{369}$	(-62.83,-61.64)	(-41.02,-39.78)
	MBD2	(-75.15,-74.11)	(-27.99,-26.80)
	MBD2-p66α	(-59.71,-59.41)	(-45.48,-45.19)
L_{383}	ABA:$MBD2_{369}$	(-64.03,-63.40)	(-40.61,-39.96)
	APC:$MBD2_{369}$	(-63.36,-62.26)	(-42.88,-41.75)
	MBD2	(-63.73,-63.35)	(-41.66,-41.28)
	MBD2-p66α	(-61.48,-61.16)	(-44.45,-44.14)

Table S4. ***T*** **test** ***P*** **vales on the backbone torsion angle summarized in table S3.** Red letters for the *P*-values less than 0.05, and blue letters for the *P*-values greater than 0.05 in the interest of study.

Residue	Φ T-test	APC:MBD2$_{369}$	MBD2	MBD2-p66α	Ψ T-test	APC:MBD2$_{369}$	MBD2	MBD2-p66α
D$_{366}$	ABA:MBD2$_{369}$	0.2856	<2.2e-16	<2.2e-16	ABA:MBD2$_{369}$	0.1096	<2.2e-16	<2.2e-16
	APC:MBD2$_{369}$	-	<2.2e-16	2.692e-11	APC:MBD2$_{369}$	-	<2.2e-16	<2.2e-16
	MBD2	-	-	<2.2e-16	MBD2	-	-	<2.2e-16
I$_{369}$	ABA:MBD2$_{369}$	0.782	2.592e-13	0.8193	ABA:MBD2$_{369}$	0.0252	2.935e-12	<2.2e-16
	APC:MBD2$_{369}$	-	1.458e-06	0.6651	APC:MBD2$_{369}$	-	1.017e-10	1.534e-06
	MBD2	-	-	<2.2e-16	MBD2	-	-	<2.2e-16
V$_{376}$	ABA:MBD2$_{369}$	0.02737	<2.2e-16	<2.2e-16	ABA:MBD2$_{369}$	1.392e-04	<2.2e-16	<2.2e-16
	APC:MBD2$_{369}$	-	<2.2e-16	<2.2e-16	APC:MBD2$_{369}$	-	<2.2e-16	<2.2e-16
	MBD2	-	-	<2.2e-16	MBD2	-	-	<2.2e-16
L$_{383}$	ABA:MBD2$_{369}$	0.005058	0.3659	<2.2e-16	ABA:MBD2$_{369}$	1.773e-09	1.044e-09	<2.2e-16
	APC:MBD2$_{369}$	-	0.01336	<2.2e-16	APC:MBD2$_{369}$	-	0.005753	9.003e-11
	MBD2	-	-	<2.2e-16	MBD2	-	-	<2.2e-16

Table S5. Primer sets for vector construction.

For vector construction		
	Forward	Reverse
peYFP-N1-MBD2	5'-GTA AGA TCT ATG CGA GCG CAC CCG GGG-3'	5'-CAT GAA TTC GGC TTC ATC TCC ACT GTC-3'
mcherry-p66α-pcDNA3	5'-GTA CTC GAG ATG ACC GAA GAA GCA TGC-3'	5'-CTA TCT AGA GTT TTC CAC GTT GCT GAC TG-3'
pRSET-dTomato-MBD2	5'-ATA GGA TCC AAT GCG AGC GCA CCC GGG G-3'	5'-CAT GAA TTC GGC TTC ATC TCC ACT GTC-3'
pRSET-eYFP-p66α$^{1-206}$	5'-GTA GGT ACC ATG ACC GAA GAA GCA TGC-3'	5'-CTA AAG CTT CTA TTT CCA CGT TGC TGA CTG-3'
