# Supplementary material for: Rational discovery of antimetastatic agents targeting the intrinsically disordered region of MBD2
Source: Sci Adv. 2019 Nov 20;5(11):eaav9810. doi: 10.1126/sciadv.aav9810 (PMC6867884; doi:10.1126/sciadv.aav9810)
Supplement: Original data file S1 [file aav9810_Original_data_file_S1.zip › aav9810_Blinded Review Report_seq1_v1_InsungNaPart_PDBfiles-Description_20190411.docx]

Figure 1D molecular docking PDB files description

1. c-Myc-10058-F4.pdb

Chain A : residue name POS is 10058-F4.

Chain B : c-Myc residues taken from PDB ID 1NKP chain A 942-977 residues.

2. MBD2-ABA.pdb

Chain A : ABA

Chain B : MBD2 residues taken from PDB ID 2L2L first model chain B 209-244 residues

3. MBD2-APC.pdb

Chain A : APC

Chain B : MBD2 residues taken from PDB ID 2L2L first model chain B 209-244 residues
